# Synthesis of Citrate-Stabilized Silver Nanoparticles Modified by Thermal and pH Preconditioned Tannic Acid

**DOI:** 10.3390/nano10102031

**Published:** 2020-10-15

**Authors:** Rita La Spina, Dora Mehn, Francesco Fumagalli, Margaret Holland, Fabiano Reniero, François Rossi, Douglas Gilliland

**Affiliations:** Joint Research Centre (JRC), European Commission, 21027 Ispra, Italy; Dora.MEHN@ec.europa.eu (D.M.); Francesco-Sirio.FUMAGALLI@ec.europa.eu (F.F.); Margaret.HOLLAND@ec.europa.eu (M.H.); Fabiano.RENIERO@ec.europa.eu (F.R.); fjr.rossi@gmail.com (F.R.)

**Keywords:** Tannic acid, Silver Nanoparticles (AgNPs), synthesis, characterization

## Abstract

Silver nanoparticles (AgNPs) may be synthesized by many different methods, with those based on the thermal reduction of silver salts by citric acid or citric acid/tannic acid being amongst the most commonly used. These methods, although widely used and technically simple, can produce particles in which the size, polydispersivity and morphology can vary greatly. In this work nearly mono-dispersed spherical AgNPs have been synthesized via a one-step reduction method by using sodium citrate and varying quantities of Tannic Acid (TA), which was thermally conditioned prior to use in the growth process. It was found that the final size can be further tailored by controlling the amount of TA and the thermal conditioning of the TA at 60 °C at different time points, which changes the size and polydispersivity of AgNPs. To better understand the origin of this effect, optical spectroscopic analysis and ^1^H NMR of the TA following mild thermal conditioning of the solution have been done. Comparison of thermally conditioned TA and TA exposed to basic pH shows that similar chemical modifications occur and consequently produce similar effects on growth when used in the synthesis of AgNPs. It is proposed that thermal preconditioning of the TA introduces either chemical or structural changes, which decrease the final particle size under a given total silver content.

## 1. Introduction

Amongst the many types of nanomaterials currently in common use, silver nanoparticles (AgNPs) form one of the largest and fastest growing categories exploited in a wide variety of commercial and scientific applications. The synthesis of such materials has been studied for many years, and a wide variety of different methods has been reported in scientific literature. For example, the synthesis of AgNPs has been reported using photo-induced [1], electrochemical [2] and solvothermal methods [3] as well as the much more common chemical (NaBH_4_) and thermal reduction in aqueous citrate solution [4]. The use of natural extracts for the synthesis of AgNPs has also been investigated. Depending on the actual size required, the methods of synthesising water-dispersible AgNPs, which are probably the simplest and most commonly adopted, are based on the reduction of silver salts in the presence of sodium citrate as a principal stabiliser/reducing agent [5].

Such methods, although widely used and desirable because of their low cost, simplicity and low toxicity, are not always suitable as they may require careful optimisation to avoid broad size distributions and mixtures of different morphologies.

For many applications, particularly those exploiting the antibacterial properties of AgNPs [6,7], the size and shape of the particles may be of secondary importance, and consequently, this puts fewer constrains on the synthesis process. In other applications, such as optical detectors [8,9] or diagnostics [10], where the unique plasmon resonance properties of AgNPs are critical, the level of reproducibility and control over size and shape required in the synthesis process may be much more stringent. In these cases, the production of particles with a specific, narrow size distribution by the simple citrate method may require extensive empirical optimization of numerous interdependent parameters (temperature, time, [Ag^+^] and [Na-citrate], pH, etc.). For size-controlled particle synthesis, the alternative strategy of a 2-step seed-regrowth method [11] may be a more flexible solution, but this still requires the production of at least one batch of nanoparticles with well-controlled sizes and shapes.

Recent studies have shown that control over basic citrate methods may be improved by the use of selected organic additives [12], which, having greater reducing capacity than citrate and inducing more rapid nucleation, lead to a smaller but narrower final size distribution as compared to citrate alone. In some cases, it has been hypothesized that the use of citrate alone may cause heterogeneous growth (wires, rods, etc.) by preferential binding to certain crystal faces, but when competing with additives, with less specific binding, homogenous growth is promoted, thus producing mainly spherical, isotropic particles.

One example of such an additive is tannic acid (TA), a polyphenol compound extracted from plants that has already been reported in literature as being suitable for the synthesis of AgNPs [13,14]. Chemically, TA is formed from a glucose core that is connected to five di-gallic acid units through five –OH groups, forming ester bonds, as shown in Figure 4. It does not contain free carboxylic groups as these are covalently bound to form ester groups which may partially hydrolyse under mild acidic/basic conditions into glucose and gallic acid units [13] and/or pyrogallol [15] units and/or may form intermediate quinone derivatives [16]. TA is also a five-armed chelator agent which can coordinate with up to 20 silver ions [17]. TA has 25 phenolic –OH groups, of which 10 pairs of o-dihydroxyphenyl groups can participate in redox reactions to form quinones and to donate electrons to reduce a silver salt to metallic silver [17].

It has been reported that the size and morphology of AgNPs depend on both the concentration of TA (1–5 mM) and the pH of the solution [13]. For instance, at pH 7, an increase in the TA concentration from 1 to 5 mM produces a change in the morphology from silver nanowires to 10–30 nm spherical nanoparticles. In contrast, at a fixed concentration of 3 mM TA, varying the solution pH from 8 to 6 produced AgNPs of several different morphologies from spherical nanoparticles to mixtures of nanowires. The pH of the reaction solution has also been reported to influence the rate of the reduction reaction with a consequent modification in the shape [13] and the size [18] of the AgNPs produced. Furthermore, TA at pH 8 was shown to be able to reduce AgNO_3_ to predominantly spherical AgNPs for which the size could be controllably varied (in the range 22.1–3.3 nm) by decreasing the molar ratio of TA/AgNO_3_ from 1 to 0.05 [17]. In the work of Dadosh [19], the synthesis of AgNPs in a Na-citrate 1.3 mM solution containing 4.7 × 10^−6^ M to 3.6 × 10^−7^ M concentrations of TA-produced AgNPs with a mean size that increased from 18 to 30 nm as the TA concentration was increased. In this work, the effect of TA on the synthesis of AgNPs was studied. In particular, a previously unexplored range of TA concentration, varying from 0 to 2 × 10^−5^ M, was considered. In addition, the effect of pretreatment of TA at 60 °C at different time points has been investigated.

In our work, nearly mono-dispersed spherical silver nanoparticles (AgNPs) were synthesized via a one-step reduction method using sodium citrate and varying quantities of TA, which was thermally conditioned prior to use in the growth process. The thermal conditioning step was initially adopted to ensure the full solubility of TA, but in early experiments, it was noted that certain parameters of the thermal conditioning process had a clear influence on both the size and the shape of the resulting AgNPs. Optical spectroscopic analysis and ^1^H NMR were used to try to better understand the modification in the TA, which occurs during mild thermal conditioning of the solution, and how it influences the synthesis of AgNPs. Finally, a comparison of the thermally conditioned TA and TA preconditioned at basic pH was undertaken and the same chemical and/or structural arrangement in the TA molecules was observed. These observations indicate that changes in the TA structure influence the synthesis of AgNPs.

## 2. Materials and Methods

### 2.1. Chemicals

Silver nitrate (AgNO_3_, 99.9999% metal basis), tannic acid (TA) and gallic acid (GA) were purchased from Sigma-Aldrich (Milan, Italy), tri-sodium citrate di-hydrate (citrate) was purchased from Merck (Milan, Italy). All the other reagents were at analytical purity and were used without further treatment. Solutions were prepared in deionized water from Millipore milli-Q water purification system. Molecular structure and purity of TA and GA were verified by ^1^H NMR using a 600 MHz Bruker Avance III HD spectrometer equipped with cryo-probe (Bruker, Ettlingen, Germany).

### 2.2. Synthesis of AgNPs via Thermal Conditioning of TA

The synthesis of silver nanoparticles was carried out by reduction of AgNO_3_ with citrate and TA using a modification of the procedure previously described by Dadosh [19]. The modification consisted of thermal preconditioning the TA and citrate solution prior to their addition to the boiling AgNO_3_ solution_._

The general procedure for the synthesis was as follows: thermally conditioned samples of TA in water were prepared by mixing 1.2 mL of appropriate stock solutions of TA (from 0 to 4.7 × 10^−4^ M) with 6 mL of an aqueous 28 mM sodium citrate solution heated to 60 °C. This mixture was then maintained at a constant temperature of 60 °C in a temperature-controlled oil bath for the desired time period (0–3 h) before being added under vigorous stirring to 94 mL of boiling AgNO_3_ (0.55 mM) held in a 100-mL round-bottom flask heated in a microwave synthesis reactor (Discover S by CEM corporation). This reaction mixture was then kept at 97 °C for a further 40 min before rapid cooling to below 40 °C by compressed air.

The use of microwave heating was to ensure uniform heating without formation of hot spots, which may influence local growth. The nanoparticles were characterized directly after synthesis.

The synthesis of AgNPs was carried out by varying the concentration of TA (0, 3.3 × 10^−7^, 3.3 × 10^−6^ and 3.3 × 10^−5^ M) and by keeping the thermal conditioning time of citrate and TA solution at 60 °C for 45 min. Moreover, AgNPs were prepared by keeping the concentration of TA at 3.3 × 10^−5^ M and 3.3 × 10^−4^ M constant while varying the thermal conditioning time of citrate and TA solution at 60 °C for 0, 15 min, 45 min, 90 min and 180 min. In all cases, the concentrations of AgNO_3_ and sodium citrate were constant, fixed at 0.55 mM and 1.4 mM, respectively.

### 2.3. Synthesis of AgNPs via pH Conditioning of TA

TA solution at concentrations of 40 µM and 400 µM were left to equilibrate overnight at pH 10. For each TA concentration, 1 mL of 0.17 M of citrate was added to 6 mL of TA solution to achieve the same concentration of chemicals as for the thermal conditioning step. After mixing the solution, 6 mL of the citrate/TA solution was rapidly added to a boiling solution of AgNO_3_ and the reaction was allowed to proceed as described above.

### 2.4. Nanoparticle Characterisation

The optical properties of silver NPs were investigated by UV-visible (UV-vis) spectroscopy (Thermo Electron Corporation, Nicolet Evolution 300, (Waltham, MA, USA). The particle size and the size distribution of synthesized AgNPs were assessed by Centrifugal Liquid Sedimentation (CLS) measurements (Disk centrifuge model DC24000UHR by CPS Inc. (Oosterhout, The Netherlands). The CLS measurements were performed in an 8 wt%–24 wt% sucrose density gradient with a rotation disc speed of 22,000 rpm. Each sample injection of 100 µL was preceded by a calibration step using Polyvinyl chloride (PVC) particle size standards with a weight mean size of 280 nm. In addition, the particle size distribution of AgNPs was also determined by dynamic light scattering (DLS) by Malvern Zetasizer Nano-ZS Ltd., (Malvern, UK). Each sample was analysed in triplicate at 25 °C after a temperature equilibration step of 120 s and with an acquisition time of 80 s. For each sample, the hydrodynamic diameter and Polydispersivity Index (PDI) were calculated by the DLS internal software. Z-potential was measured using the same instrument and was recorded in a DTS1060C disposable cell with an equilibration time of 120 s. Measurements were performed just after measuring the pH of the NP solution. Moreover, the size distribution and morphology of the AgNPs were analysed by Transmission Electron Microscopy (TEM) (JEOL 2100, Tokyo, Japan) at an accelerating voltage of 200 kV. TEM analysis of the AgNPs was performed by spotting 5 µL of solution onto ultrathin Formvar-coated 200-mesh cupper grids (Ted Pella Inc., Redding, CA, USA) and left to dry in air at 4 °C. TEM images were then analysed with ImageJ, Java-based image processing program, (to obtain the shape factors (Roundness), average size and the size distribution of at least 200 particles for each sample. The shape factor of the AgNPs is calculated by roundness = 4 × area/(π (major axis)^2^), where the major axis means the major axis of the particle’s fitted ellipse. AgNP thin films drop casted on soda-lime glass were analysed with no preliminary treatments with an X-ray diffractometer operated in Bragg-Brentano mode (D8 Advance, Bruker AXC GmbH, Karlsruhe, Germany). A 0D-LYNXEYE detector was selected to carry out the measurements. Current and voltage were set at 40 mA and 40 kV, respectively. Göbel mirror, 0.6-mm slit and 2.5° Soller were inserted along the primary beam path. A notch knife was used to avoid saturating the detector counts at small angles. A 0.6-mm slit and 2.5° Soller were mounted on the secondary beam path. In a typical scan, 2θ ranged from 35 to 85°, step size was 0.025° and time per step was 15 s.

### 2.5. Characterisation of TA Solution

The role of TA in synthesis of AgNPs was investigated by collecting aliquots of a TA solution preheated at 60 °C for different time periods and characterised immediately by UV-vis spectroscopy and ^1^H NMR. In addition, the TA solutions were left to equilibrate at several pH values and then characterised by UV-vis spectroscopy and ^1^H NMR after 24 h. The NMR experiments were performed at 300 K on a Bruker (Rheinstetten, Germany) spectrometer Avance III HD 600 (nominal proton frequency 600.13 MHz) equipped with a 5 mm QCI cryo-probe. Measurements were made in water with the addition of 10% deuterated water for the frequency locking signal. The ^1^H chemical shifts are expressed in δ scale (ppm), referenced to the proton signal of the Trimethylsilylpropanoic acid (TSP) (0.0 ppm for proton). Compounds were characterised by one-dimensional (1D) NOESY presaturation experiments, which are used for the suppression of the dominant water signal.

## 3. Results

### 3.1. Effect of TA Concentration

The synthesis of all AgNPs was performed by setting the concentration of AgNO_3_ at 0.55 mM and the concentration of citrate at 1.4 mM; after synthesis, the pH of the colloidal solution was around 6.5. As the first stage, AgNPs were synthesized by varying the concentration of TA in citrate solution (0, 3.3 × 10^−7^, 3.3 × 10^−6^ and 3.3 × 10^−5^ M). In each case, the TA solution was mixed with the citrate solution and then thermally preconditioned at 60 °C for 45 min prior to addition to a boiling solution of AgNO_3_.

**Figure 1:**
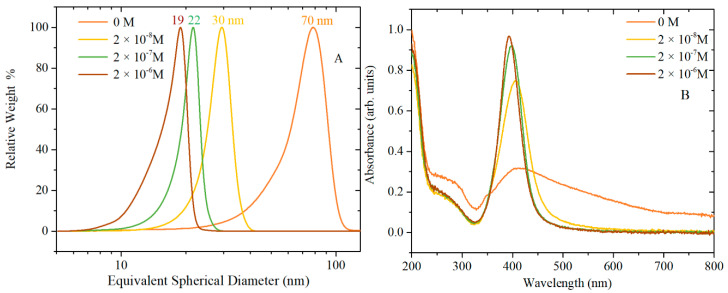
(**A**) Centrifugal Liquid Sedimentation (CLS) results and (**B**) UV-vis spectra of Silver nanoparticles (AgNPs) synthesised at various concentrations of Tannic Acid (TA): 0 M, 2 × 10^−8^, 2 × 10^−7^ and 2 × 10^−6^ M. CLS and UV-vis spectra show that increasing the TA concentration produces smaller and less polydispersed AgNPs.

**Figure 2:**
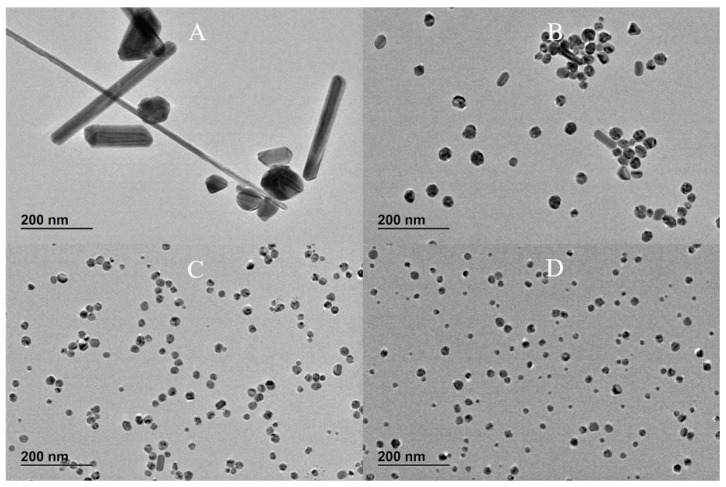
TEM images of AgNPs synthesised at various TA concentrations: (**A**) 0 M, (**B**) 2 × 10^−8^ M, (**C**) 2 × 10^−7^ M and (**D**) 2 × 10^−6^ M.

Figure 1 shows CLS particle size distributions, UV-vis absorption and morphology of AgNPs caused by varying the concentration of TA, while Figure 2 shows the corresponding TEM images. Table S1 shows the summary of NPs size and size distribution through different techniques (CLS, DLS and TEM), zeta potential characterization, pH of the TA/citrate solution prior to addition to boiling solution of AgNO_3_, and finally, the pH of the AgNPs colloidal solution.

The UV-vis spectra in Figure 1B show the surface plasmon resonance (SPR) band, which is influenced by the particles’ shape and size, and by the polydispersivity of the nanoparticles. When no TA is added to the synthesis solution, the SPR band is broad and absorption is observed also at wavelengths greater than the peak at 413 nm. This broad SPR band is the result of large spherical as well as nonspherical particles, which is normally associated with broadening and red-shift of the SPR band. The CLS data of these nanoparticles show a wide peak which is characteristic of a polydispersed size distribution of nanoparticles; DLS data (Table S1 and Figure S1) and the TEM images confirm that the synthesis of AgNPs by citrate reduction without any TA added to the synthetic solution results in a polydispersed product containing also a variety of nonspherical nanoparticles. With the increasing amount of TA from 2 × 10^−8^ to 2 × 10^−6^ M, the SPR band at about 400 nm becomes narrower and sharper (the tail at higher wavelengths decreases), which is characteristic of monodispersed spherical nanoparticles. In addition, the CLS data show that the increasing amount of TA produces AgNPs with slightly narrow size distribution (sharper peak) and a shift toward lower size (lower wavelength of the surface plasmon absorption peak centre in UV-vis data). This trend is confirmed by the TEM images, which show a reduction in the quantity of rods as the concentration of TA is increased in the reaction solution. Hence, the TA has an important role in the final size, size distribution and shape of AgNPs even when such small amounts of TA are added to the chemical reaction.

X-ray diffraction analysis has been performed over a selection of synthetized materials (see data in Figures S2–S4), varying the TA concentration and thermal pretreatment time at 0 and 45 min. Analysis confirms that AgNPs are silver face-centred cubic, and their overall crystallinity is around 72 ± 6%. Samples synthetized in the presence of tannic acid exhibit an (111) facet crystallite size of 16.8 nm, while the average crystallite size of other reflections is 4.8 ± 1.4 nm. No clear dependence on preconditioning temperature and tannic acid concentration was observed on AgNP crystallite growth. Samples synthetized without tannic acid shows larger (about factor 2) crystallite size.

Dadosh [19] studied the synthesis of AgNPs using TA in the concentration range from 3.6 × 10^−7^ M to 4.7 × 10^−6^ M finding that the size of AgNPs increased with the TA concentration. A comparison of the *D_TEM_* size obtained with [TA] = 2 × 10^−7^ M in this work (20 nm) with that obtained by Dadosh at 3.6 × 10^−7^ M (18 nm) shows a good correspondence. However, the same correlation of size is not observed at [TA] = 2.0 × 10^−6^ M; in this work, *D_TEM_* was 16 nm, whereas in the Dadosh study, 2.36 × 10^−6^ M TA generated 27 nm AgNPs. The difference in these outcomes inspired the investigation of the role of TA in the synthesis of AgNPs as a function of TA concentration and preconditioning time at 60 °C. In Table 1, the experimental conditions (TA and citrate concentrations, temperature and reaction time) and the size of the obtained AgNPs synthesised in this work are compared with the conditions followed by Dadosh [19]

**Figure 3:**
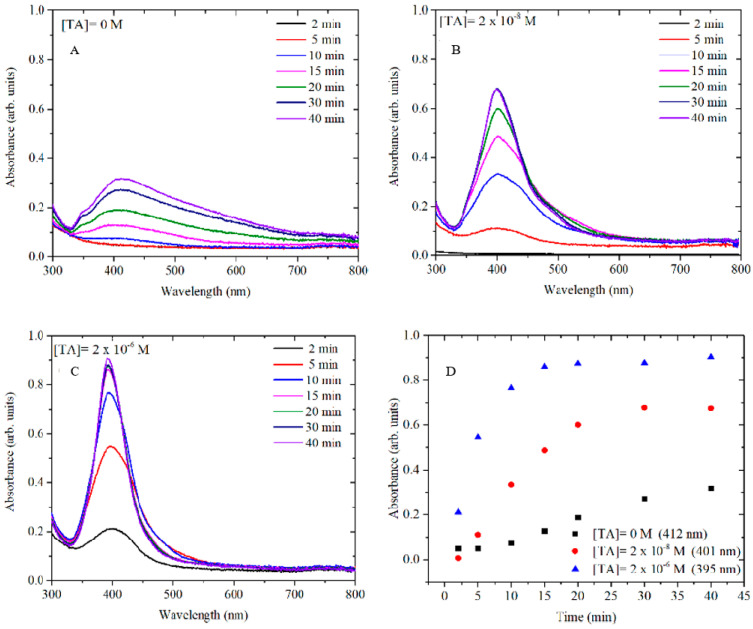
UV-vis spectra show the kinetics of AgNP growth by varying the concentration of TA: (**A**) 0 M, (**B**) 2 × 10^−8^ M, (**C**) 2 × 10^−6^ M and (**D**) plot of the kinetics at maximum adsorption for the three concentrations of TA.

In order to better understand the mechanism of TA in AgNP formation, the growth of AgNPs at different concentrations of TA (0 M, 2 × 10^−8^ M and 2 × 10^−6^ M) was monitored in time by UV-vis. The SPR band of the metallic silver was monitored at different growth times (Figure 3) while varying the concentration of TA. All samples were monitored for up to 60 min, but in all cases, the spectra remained unchanged after 40 min.

It is well known that the size and the shape of NPs depend on the induction time, the nucleation-dominant formation and the growth period [20]. Generally, a slow nucleation rate (large induction time) favours the formation of large size nanoparticles. In Figure 3**,** the growth kinetics of AgNPs at several concentrations of TA show induction times that become shorter as the concentration of TA increases. When no TA is added to the reaction (Figure 3A), the nucleation rate is slow and the induction time is over 10 min, which suggests that the formation of AgNPs may be dominated by the Ostwald ripening and coalescent attachment [21]. Hence, in this case, polymorphic and polydispersed AgNPs were obtained. In contrast, when even low concentrations of TA (sub-stoichiometric with respect to [Ag^+^]) were used in the AgNP synthesis, the induction time was reduced to 5 min when [TA] = 2 × 10^−8^ M and then to less than 2 min when [TA] = 2 × 10^−6^ M. This effect is probably due to the stronger reducing power of TA compared to citrate [22], resulting in a more rapid and uniform formation of metal clusters which coalesce into seed particles. Successively, after the TA is consumed, these seeds serve as templates for the deposition of the remaining free silver ions through a slower reduction by citrate at the particles surface. This hypothesis is supported by the observation that, as the concentration of TA increases, the size of AgNPs decreases. Therefore, a larger number of AgNPs have been formed for a large TA concentration keeping constant the amount of Ag^+^.

The observation that the presence of TA reduces the formation of nonspherical particles may be attributed to competition between TA and citric acid for absorption at different crystal growth planes. It has been reported that citric acid preferentially absorbs on the (111) face [23] of a silver crystal effectively passivating this surface and resulting in a preferential growth of the (100) surfaces, leading to the occurrence of cubic and elongated cubic structures. In our system, we observe that, upon the addition of TA at all tested concentrations, an enhancement of (111) facet growth at the expense of (200) planes (see Figure S2), which indicates the presence of isotropic AgNPs with truncated octahedron shape (dominant (111) facets) and, at the same time, the overall crystallite size decreases for all observed crystal planes (see Figure S3). These observations are compatible with the interpretation that the presence of TA, with its many hydroxyl groups, results in additional competing passivation of the (100) face, or alternatively, stronger nonspecific binding by TA may cover all faces of the silver, thereby impeding absorption of the citrate and thus inhibiting oriented growth.

### 3.2. Synthesis of AgNPs by Thermal Preconditioning of TA

**Figure 4:**
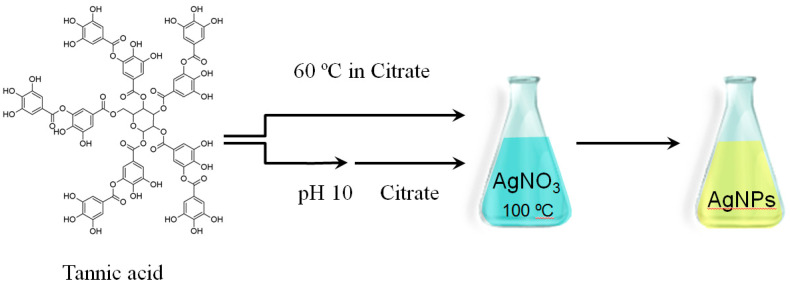
Synthesis of AgNPs by thermal preconditioning of TA in citrate at 60 °C from 0, 15, 45 and 90 to 180 min and by pH preconditioning of TA at pH 10: the TA/citrate solution was then added to a boiling solution of AgNO_3_, and AgNPs were formed.

In order to understand what is occurring during TA preconditioning, the synthesis of AgNPs was performed following two different synthetic routes (Figure 4). In the first route, the TA/citrate solution was preconditioned at 60 °C from 0, 15, 45 and 90 to 180 min and then added to a boiling solution of AgNO_3_. In the second route, TA was preconditioned for 24 h at pH 10, without heating, and mixed with a citrate solution before finally being added to a boiling solution of AgNO_3_. The influence of both routes on the AgNPs final product was explored at final TA concentrations of 2 × 10^−6^ and 2 × 10^−5^ M in the AgNPs solution. The concentrations of AgNO_3_ and citrate were kept constant throughout the synthesis.

**Figure 5:**
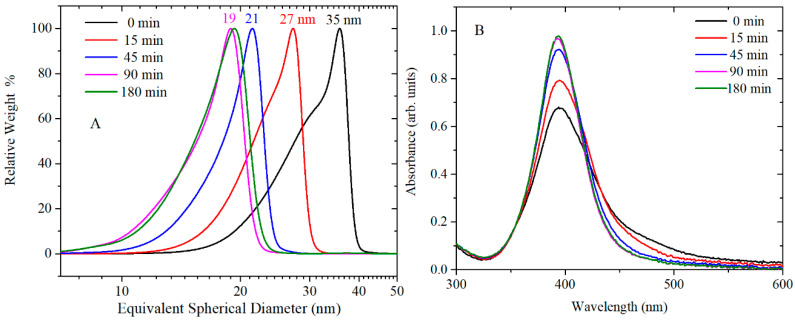
(**A**) CLS results and (**B**) UV-vis spectra of AgNPs synthesised using a fixed concentration [2 × 10^−6^ M] of TA solution thermally preconditioned for different times: 0, 15, 45, 90 and 180 min. CLS and UV-vis spectra show that, by increasing the preconditioning time of TA, smaller sizes and narrower size distributions of AgNPs were obtained.

**Figure 6:**
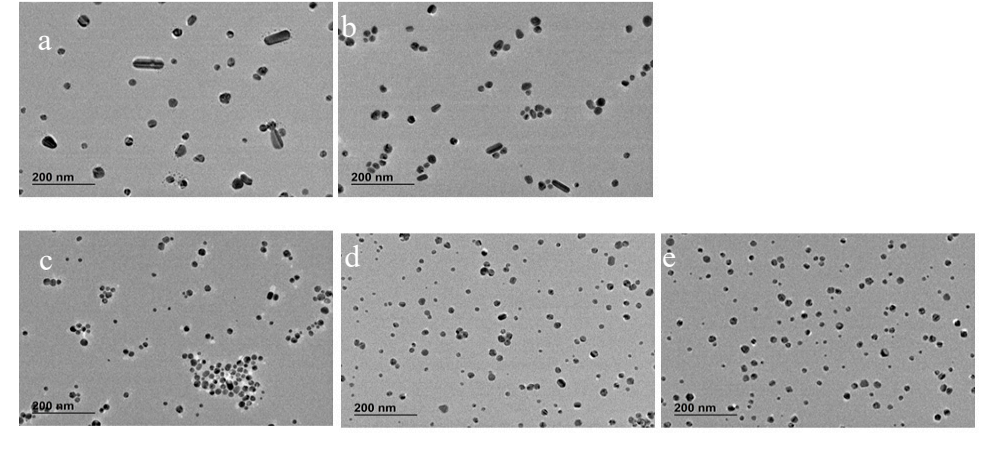
TEM images of AgNPs synthesised using TA solution thermally preconditioned for (**a**) 0 min, (**b**) 15 min, (**c**) 45 min, (**d**) 90 min and (**e**) 180 min.

Figure 5 and Figure 6 show the CLS, UV-vis and TEM images of AgNPs synthesised by reduction of AgNO_3_ with citrate and TA solution. TA was thermally preconditioned in citrate solution at 60 °C for 0, 15 min, 45 min, 90 min and 180 min. The concentration of TA in AgNPs solution was 2 × 10^−6^ M, and after synthesis, the pH of AgNPs colloidal solution was pH 6.5. At larger thermal preconditioning time, the UV-vis spectra showed a sharper SPR band at 400 nm and the tail at 500 nm became less pronounced; this trend is associated to mono-dispersed spherical nanoparticles. In addition, the number of rod-shaped nanoparticles decreases as confirmed by TEM image. No clear effect on crystallization properties was observed in X ray diffractograms (see Figure S4) of AgNPs synthesised with and without 45 min thermal preconditioning. The CLS data show a shift toward smaller nanoparticle size as the thermal preconditioning time at 60 °C increases; hence, the thermal preconditioning time of TA in citrate solution at 60 °C influences the shape, the size and the size distribution of AgNPs. As additional confirmation, the size of AgNPs was determined by DLS (Table S2 and Figure S5). However, the results were not easy to interpret for these samples because this technique is not well adapted to polydispersed and nonspherical shaped samples. Evaluation of the particle shape from TEM images using ImageJ software showed that the roundness of the AgNPs slightly improved from 0.81 ± 0.14 to 0.85 ± 0.09 by using TA thermal preconditioning from 0 to 180 min. For these samples, the measured magnitude of the Z-potential was found to increase slightly from −47 to −37 mV, as more TA was added but, overall, remained sufficiently high to maintain colloidal stability through electrostatic repulsion.

A series of similar experiments were performed using the same concentration of AgNO_3_ and citrate as used previously but with the TA concentration being increased by a factor of 10 to a value of 2 × 10^−5^ M. As in the previous case, TA was thermally preconditioned in citrate for 0, 15, 45, 90 and 180 min and, after synthesis, the pH of the AgNPs colloidal solution was around 7. The CLS and UV-vis results are shown in Figure S6 and the corresponding TEM pictures in Figure S7.

In this case, the TEM images show that NPs with high roundness were obtained at each preconditioning time. Not only the shape but also the size of the AgNPs appeared to be independent of the thermal preconditioning step up to 90 min as confirmed by CLS and DLS data (Table S3). Surprisingly, a further increase in the thermal pretreatment to 180 min produced a slight decrease in the size of AgNPs, from 24 to 20 nm, accompanied by a drastic narrowing in the size distribution of AgNPs. DLS data (Table S3 and Figure S8) show that the PDI goes from 0.311 (zero preconditioning time of TA) to 0.05 (after 180 min of TA preconditioning time), indicating mono-dispersed and round-shaped NPs. A change in the Z-potential of the samples change from −47 to −43 mV again confirms the electrostatically stabilised nature of the AgNPs suspension. Summarising, the synthesis of AgNPs by thermal preconditioning of TA at a concentration of 2 × 10^−5^ M in the synthesis mixture produced close to spherical AgNPs with a size dependence only being observed with extended (180 min) thermal preconditioning.

### 3.3. Effect of the Thermal Conditioning on TA

**Figure 7:**
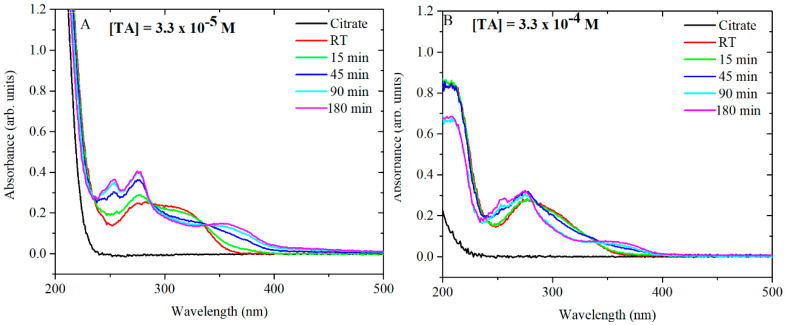
UV-vis spectra of TA thermally preconditioned at 60 °C for various times in 21 mM citrate: (**A**) TA 3.3 × 10^−5^ M and (**B**) TA 3.3 × 10^−4^ M. For the purpose of comparison, the spectra were obtained from thermal conditioned samples which, immediately prior to UV-vis analysis, were diluted in water to obtain a TA concentration of 4 × 10^−6^ M. In the UV measurements, the concentration of citrate is 2.5 mM in (**A** and 0.25 mM in **B**).

For the synthesis of AgNPs, TA is equilibrated in citrate at 60 °C and then added to a boiling solution of AgNO_3_. As mentioned previously, TA plays an important role in the formation of AgNPs with specific shape, size and size distribution. Consequently, further studies were performed to focus on the behaviour of TA during the thermal preconditioning step. Firstly, TA-citrate solutions at pH 7 were analysed by UV-vis in order to understand the behaviour of TA after being heated up at 60 °C. TA-citrate aliquots were collected at different time points. The tested TA concentrations were 3.3 × 10^−5^ M and 3.3 × 10^−4^ M. Note that these two concentrations when diluted (6 mL in 94 mL of AgNO_3_) give the desired final concentrations (2 × 10^−6^ and 2 × 10^−5^, respectively) of TA in the synthesis mixture (Figure 7A,B). At TA concentrations of 3.3 × 10^−5^ M, the UV spectra showed a radical shift of the absorbance peaks already after 15 min of thermal preconditioning. The same trend was detected for 3.3 × 10^−4^ M of TA, although in this case, the complete shift of the peaks occurred only after 180 min at 60 °C. It has been already shown that citrate makes TA oxidation most readily, probably as a result of hydrogen bonding interactions between the hydroxyl groups of tannic acid and the oxygen atoms of carbonyl groups of citrates [14]. Additional experiments were performed in water at pH 5.8 and in phosphate-buffered saline (PBS) at pH 7 in order to determine whether the temporal changes in the UV-vis spectra of the TA were due to the interaction of TA with citrate and/or to the pH of the solution.

The UV-vis spectra of TA in PBS (Figure S9A,B) and in water (Figure S9C,D) heated up to 60 °C at different time points are shown. The solutions of TA at the concentration of 3.3 × 10^−5^ M in PBS and preincubated at 60 °C were monitored by ^1^H NMR at different time points to study the modification of the chemical shift (Figure S10). It is observed that the peak at 7.0 ppm can be assigned to the tannic acid in the hydroquinone form. During preconditioning, the peak at 7.0 ppm disappears and a peak at 7.5 ppm appears, which can be assigned to the quinone derivatised form of tannic acid [24] 

**Figure 8:**
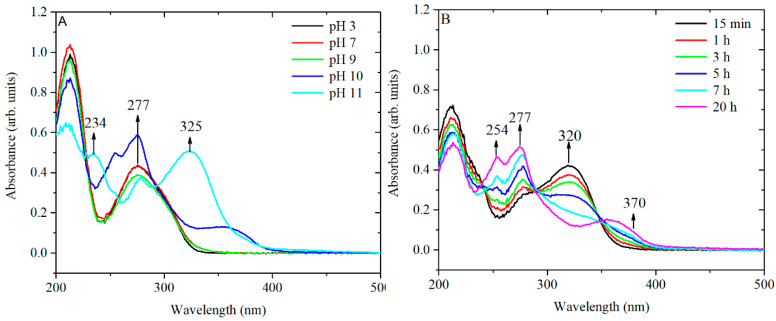
(**A**) UV-vis spectra of TA solutions equilibrated for 24 h at various values of pH and (**B**) temporal variation of the UV-vis spectra of TA at pH 10 at room temperature (RT).

The spectra showed that, at pH 5.6 in water, the TA (Figure S9C,D) appears to be stable, while under more basic conditions in PBS and Na-citrate, there is a clear modification in the composition of the solution. The similarity of the behaviour with and without citrate suggests that the changes are not directly linked to the presence of citrate itself but to its effect on the pH. In addition, the influence of pH was further confirmed in similar experiments (Figure 8) in which TA was equilibrated for 24 h at 20 °C in an aqueous solution in which the pH had been modified by the addition of small amounts of HCl or NaOH.

The obtained spectra confirm that strong basic conditions can induce chemical changes without additional heating while, under neutral or moderately basic conditions (pH 7–8), these chemical changes require input of additional heat to produce an effect in a reasonable time.

The relevance of these results can be seen considering the results on the behaviour of TA under several pH conditions [16,25]. The peaks at around 220 and 277 nm are associated with the neutral form of TA (from pH 2 to pH 8), whereas in strong basic conditions (pH 11), two additional peaks appear at approximately 234 and 325 nm, which are assigned to the ionized phenolic groups of TA. The ratio of the peaks A_276_/A_325_ has been used to monitor the concentration of ionized phenolate groups. The peaks at 254 and 370 nm (at pH 10) are most likely related to the rearrangements of an ionized structure of TA to various conjugated quinone derivatives [26,27]. ^1^H-NMR spectra of TA in PBS at 60 °C confirmed the decreased intensity of the peaks related to the hydroquinone form of TA and an increase in the intensity of the peak related to the quinone form. Similar chemical shifts are observed in TA equilibrated at pH 10 (Figure S10).

**Figure 9:**
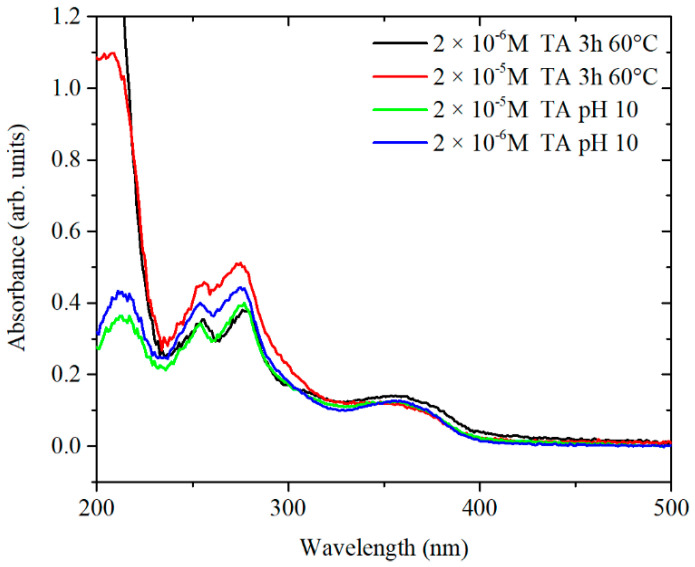
Comparative UV-vis spectra of TA thermally preconditioned at 60 °C and equilibrated overnight at pH 10.

To confirm the hypothesis of chemical rearrangements inside the TA structure, a solution of TA of 3.3 × 10^−4^ M was equilibrated at pH 10 and the kinetics of peak shifting was monitored over 24 h (Figure 8B). The presence of three isosbestic points at 238, 289 and 350 nm suggests that at least three different forms of TA, convertible from one to the other, might be present in solution. Overall, the UV-vis spectrum of TA equilibrated at pH 10 for 24 h and the spectrum of TA preconditioned at 60 °C, at pH 7.4 for 3 h are similar. Therefore, it is likely that the same chemical species are formed by treating the TA in both ways, as shown in Figure 9.

Earlier, the behaviour of TA under thermal treatment in a closed reactor was reported [15] describing the thermal hydrolysis of TA to Gallic acid (GA) and pyrogallol. Significant amounts of GA were recorded at 150 °C, whereas at 200 °C, the production of pyrogallol reached a maximum. At 60 °C, pyrogallol was not detected and no significant amount of GA was observed either [15]. In order to test whether in our experimental system any GA was produced by thermal preconditioning of TA, GA was heated up at 60 °C for 0 15, 45, 90 and 180 min in citrate and in PBS (Figure S11). The UV-vis spectra of GA in citrate and PBS show a peak at 261 nm and a shoulder at 336 nm in which the absorbance increased after 15 min at 60 °C. However, the position of the absorbance peaks for TA and GA are different and confirmed no GA formation as a hydrolysis product of TA. Therefore, the changes of UV-vis spectra in time might be associated with a chemical and/or structural rearrangement inside TA molecules as a combination of pH and heat.

Furthermore, the comparison of the UV-vis spectra for TA at concentrations of 3.3 × 10^−5^ M and 3.3 × 10^−4^ M show a difference in the kinetics of the chemical and/or structural rearrangement. In detail, the UV-vis spectrum of 3.3 × 10^−5^ M TA after 45 min at 60 °C is much more similar to the UV-vis spectrum of TA equilibrated at pH 10. 5 than TA at 3.3 × 10^−4^ M. In the latter case, the changes of UV spectra were less pronounced in time and this suggests that more than one mechanism might be involved. The only difference between the two solutions was the concentration of TA, which suggests that rearrangements in TA molecules are time- and concentration-dependent. A possible explanation for this behaviour may come from the polyphenolic structure of TA, which, through hydrogen bonding, may produce stable structures hindering the structural rearrangement of TA [25]. The behaviour of the TA was explored to understand the effect of pH and the thermal conditioning on the synthesis of AgNPs. The results show that, in diluted condition, 2 × 10^−6^ M TA leads to the formation of stabilised quinone derivatives that have a dominant role in the subsequent particles growth. The nature of this change in growth is not fully understood, but a number of possible hypotheses may be taken into consideration to explain the effect of smaller particles and uniformity in size resulting from the thermal preconditioning of TA. A possible hypothesis is that TA molecules form a complex with a portion of Ag^+^, leaving silver in two forms: free and bound to TA. In this condition, rapid reaction of free Ag^+^ ions form nuclei whereas the subsequent growth of the particles is slower from bound silver, which might lead to a thermodynamic control of the reaction.

Another hypothesis is that the absorption of TA on the silver particle surface leads to blocking of growth and moving toward thermodynamic control.

This statement is in agreement with the data presented in Figure 5A, where the size of the particles decreases as the time of preconditioning goes from 15 min to 90 min. In addition, Figure 7A shows increased formation of stabilised quinone derivatives after 45 min, which correspond to a drastic decrease in the size and polydispersivity of AgNPs (Figure 5A). A different mechanism might lead the formation of AgNPs at the concentration of 2 × 10^−5^ M TA. At this concentration, TA is strongly affected by the heat and preconditioning time after 180 min (Figure 7B), and only in that situation, AgNPs presented strong size dependence (Figure S6). In this case, TA is in neutral form until 45 min of preconditioning time (Figure 7B) and no major difference can be observed in the size of AgNPs. These data suggest that the main role of TA in the synthesis of AgNPs is the reduction of Ag^+^ by TA. In this case, the combination of citrate and TA increase the nucleation of AgNPs leading to an average TEM size of 24–25 nm. After 180 min of preconditioning, the mechanism of nucleation change and the presence of stabilised quinone derivated might influence the formation of seeds and the growth of the AgNPs, giving an average TEM size of 19 nm.

### 3.4. Synthesis of AgNPs by pH Preconditioning of TA

**Figure 10:**
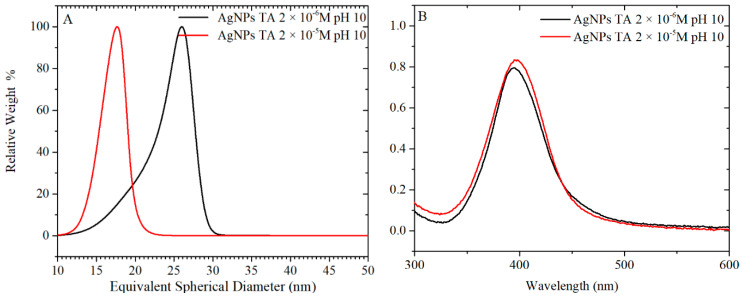
(**A**) CLS and (**B**) UV-vis of AgNPs synthesised by preconditioning TA at pH 10 for 24 h: TA were used at concentrations of 2 × 10^−6^ and 2 × 10^−5^ M.

**Figure 11:**
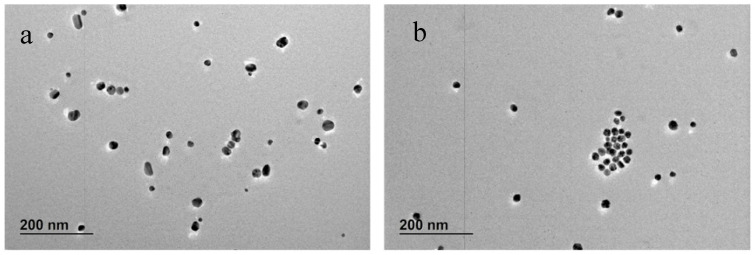
TEM images of AgNPs synthesised by preconditioning the TA at pH 10 for 24 h: the two concentrations of TA were (**a**) 2 × 10^−6^ and (**b**) 2 × 10^−5^ M.

For the synthesis of AgNPs, 40 and 400 µM of TA were equilibrated at pH 10 overnight. The TA solution was mixed with a concentrated solution of citrate and then, immediately added to the boiling solution of AgNO_3_. The final concentrations of TA in the AgNPs suspension were, as in the previous case, 2 × 10^−6^ and 2 × 10^−5^ M. The UV-vis spectra and TEM images of particles formed using these TA solutions are plotted in Figure 10 and Figure 11, respectively, while a more complete summary of the AgNPs characterisation is reported in Table S4 and Figure S12. The results show that AgNPs of similar size and size distribution are obtained either by thermal preconditioning or by pH preconditioning of TA at pH 10. In particular, 2 × 10^−6^ TA produces particles of 17 ± 5 nm D*_TEM_* by thermal preconditioning and 21 ± 6 nm D*_TEM_* by pH preconditioning, whereas 2 × 10^−5^ TA produces particles of 19 ± 3 nm D*_TEM_* thermal preconditioning and 19 ± 3 nm D*_TEM_* by pH preconditioning. These results support the theory that the same chemical rearrangement of the TA can be obtained by two different experimental routes: treating the TA at 60 °C or equilibrating it at pH 10.

Overall, the use of a thermal rather than a pH-based preconditioning step has been shown to be an effective means to better control the size and polydispersivity of the final product. These results show that the presence of TA in the quinone derivated form is obtained in basic pH and preconditioning time at 60 °C. In addition, the TA in the quinone derivated might influence the formation of seeds and the growth of AgNPs.

## 4. Conclusions

The results show a time-dependent chemical modification of TA which, in the absence of other changes in the AgNPs synthesis conditions, appear to have a strong influence on the final distribution of particle size and, to a lesser extent, the shape.

Our findings demonstrate that the addition of even low concentrations of the thermally conditioned TA greatly decreases the mean particle size, improves polydispersivity and suppresses the formation of nonspherical particles. It was found that the final size can be further tailored by controlling the amount of TA and the degree of chemical structure rearrangement occurring during TA thermal conditioning. In addition, it was found that the extent of the chemical rearrangement induced by a short (time < 3 h) thermal treatment was comparable to that resulting from preconditioning for 24 h at an elevated pH.

Finally, the combination of the pH buffered citrate solution and the heating time at 60 °C produced a time-dependent structural rearrangement of the TA, which controlled the size, size distribution and, more interestingly, the morphology of AgNPs. 

## Figures and Tables

**Table 1:** Comparison of experimental conditions and size of AgNPs.

Dadosh [19]	[TA] (M)	[Citrate] (M)	T (°C)	Reaction Time (min)	D*_TEM_* ± σ*_TEM_* (nm)
	3.60 × 10^−7^	1.4 × 10^−3^	60 and 100	~3 + 20	18 ± 2
	5.80 × 10^−7^	1.4 × 10^−3^	60 and 100	~3 + 20	19.5 ± 3
	1.18 × 10^−6^	1.4 × 10^−3^	60 and 100	~3 + 20	25.4 ± 2.8
	2.36 × 10^−6^	1.4 × 10^−3^	60 and 100	~3 + 20	26.6 ± 3.2
	3.52 × 10^−6^	1.4 × 10^−3^	60 and 100	~3 + 20	28.3 ± 4
	4.70 × 10^−6^	1.4 × 10^−3^	60 and 100	~3 + 20	29.7 ± 4.3
This work					
	0	1.4 × 10^−3^	100	40	98 ± 53
	2 × 10^−8^	1.4 × 10^−3^	100	40	30 ± 8
	2 × 10^−7^	1.4 × 10^−3^	100	40	20 ± 5
	2 × 10^−6^	1.4 × 10^−3^	100	40	16 ± 5

## Supplementary Materials

The following are available online at https://www.mdpi.com/2079-4991/10/10/2031/s1. Section S1: Characterisation of AgNPs. Figure S1: DLS size distribution of AgNPs synthesised by varying the concentration of TA; Figure S2: X ray diffractograms of AgNP prepared with different TA concentrations; Figure S3: Crystallite size for (111) and (200) facets (left *Y*-axis) as derived from Sherrer’s equation and their ratio (right *Y*-axis); Figure S4: X-ray diffractograms of AgNPs prepared with 2 × 10^−5^ M of TA, which was preconditioned for 45 min; Table S2: Characterisation of AgNPs synthesised by varying the preconditioning time of TA at 60 °C: the concentration of TA is 2 × 10^−6^ M; Figure S5: DLS size distribution of AgNPs synthesised by varying the preconditioning time of TA at 60 °C: the concentration of TA is 2 × 10^−6^ M; Figure S6: (A) CLS size distribution and (B) UV-vis spectra of AgNPs synthesised with 0.55 mM AgNO_3_ and 2 × 10^−5^ M of TA concentration by varying the thermal preconditioning time of TA in citrate at 60 °C for 0, 15, 45, 90 and 180 min; Figure S7: TEM pictures of AgNPs synthesised with 0.55 mM AgNO_3_ and 2 × 10^−5^ M of TA concentration, varying the thermal preconditioning time of TA in citrate at 60 °C for (A) 0 min, (B) 15 min, (C) 45 min, (D) 90 min and (E) 180 min; Table S3: Characterisation of AgNPs synthesised by varying the preconditioning time of TA at 60 °C: the concentration of TA is 2 × 10^−5^ M; Figure S8: DLS size distribution of AgNPs synthesised by varying the preconditioning time of TA at 60 °C: the concentration of TA is 2 × 10^−5^ M; Figure S9: UV-vis spectra of TA solution equilibrated at 60 °C at different time points in phosphate-buffered saline (PBS) and water: the concentration of TA is A) 3.3 × 10^−5^ M in PBS, B) 3.3 × 10^−4^ M in PBS, C) 3.3 × 10^−5^ M in water and D) 3.3 × 10^−4^ M in water; Figure S10: ^1^H-NMR (zoom of the aromatic part of the spectrum): comparative spectra of TA in PBS by varying the preconditioning time of TA at 60 °C. The concentration of TA is 2 × 10^−6^ M. Spectra of TA were equilibrated at pH 10; Figure S11: UV-vis spectra of Gallic acid (GA) preconditioned at 60 °C at different time points in A) PBS and B) citrate; Figure S12: DLS size distribution of AgNPs synthesised by pH conditioning of TA at pH 10: the concentration of TA is 2 × 10^−6^ M and 2 × 10^−5^ M. Table S1: Characterisation of AgNPs synthesised by varying the concentration of TA. Table S2: Characterisation of AgNPs synthesised by varying the preconditioning time of TA at 60 °C: the concentration of TA is 2 × 10^−6^ M. Table S3: Characterisation of AgNPs synthesised by varying the preconditioning time of TA at 60 °C: the concentration of TA is 2 × 10^−5^ M. Table S4: Characterisation of AgNPs synthesised by pH conditioning of TA at pH 10: the concentration of TA is 2 × 10^−6^ M and 2 × 10^−5^ M.
