# Supplementary material for: Synthesis of Citrate-Stabilized Silver Nanoparticles Modified by Thermal and pH Preconditioned Tannic Acid
Source: Nanomaterials (Basel). 2020 Oct 15;10(10):2031. doi: 10.3390/nano10102031 (PMC7602624; doi:10.3390/nano10102031)
Supplement: Supplementary file 1 [file nanomaterials-10-02031-s001.pdf]

Supplementary Materials: Synthesis of Citrate Stabilized Silver Nanoparticles Modified by Thermal and pH Preconditioned Tannic Acid

Rita La Spina, Dora Mehn, Francesco Fumagalli, Margaret Holland, Fabiano Reniero, François Rossi and Douglas Gilliland

S1. Characterisation of AgNPs

The characterisation of size and size distribution of Silver nanoparticles (AgNPs) by varying the concentration of Tannic acid (TA) are listed in Table S1. The characterisation of size and size distribution of AgNPs by varying the thermal conditioning time of TA at 60 °C at a concentration of 2×10^{-6} M TA is presented in Table S2 and at a concentration of 2×10^{-5} M in Table S3. The characterisation of size and size distribution of AgNPs prepared by preconditioning TA at pH 10 are listed in Table S4. The concentration of TA in the AgNPs solutions was 2×10^{-6} M and 2×10^{-5} M.

Centrifugal Liquid Sedimentation (CLS) was used to measure the Stokes particle size and size distribution, while dynamic light scattering (DLS) was used to determine the mean hydrodynamic diameter of the particle in solutions and the particle surface charge (zeta-potential). Size and shape analysis were also performed using Transmission Electron Microscopy (TEM). The pH value of the TA solutions prior to their addition to $AgNO_3$ boiling solution and the final pH of AgNPs are also listed below.

Table S1. Characterisation of AgNPs synthesised by varying the concentration of TA.

[TA]	0 M	2×10^{-8} M	2×10^{-7} M	2×10^{-6} M
$d_{CLS} \pm \sigma_{CLS}$ (nm)	70.7 ± 9.5	28.3 ± 2.7	23.5 ± 1.8	21.7 ± 2.0
$DI_{CLS} \pm \sigma_{DI}$	1.79 ± 0.55	1.15 ± 0.04	1.13 ± 0.04	1.14 ± 0.03
$d_{DLS} \pm \sigma_{DLS}$ (nm)	75.58 ± 0.28	16.32 ± 0.50	21.83 ± 0.88	18.67 ± 0.10
$PDI_{DLS} \pm \sigma_{PDI}$	0.213 ± 0.005	0.572 ± 0.012	0.339 ± 0.004	0.312 ± 0.001
$D_{TEM} \pm \sigma_{TEM}$ (nm)	98 ± 53	30 ± 8	20 ± 5	16 ± 5
Z pot± σ_Z (mV)	−53.7 ± 0.7	−48.1 ± 1.2	−44.3 ± 1.2	−36.1 ± 1.0
pH TA in citrate	7.83	7.74	7.7	7.54
pH AgNPs	6.68	6.54	6.53	6.53

d_{CLS} (nm) represents the position of the peak maximum of the AgNPs size based on weight distribution, calculated by CLS; σ_{CLS} (nm) is the standard deviation of three independent measurements by CLS calculated at Half Height Width (HHW) of the main peak. DI_{CLS} is the CLS polydispersity index (expressed as the ratio D_w/D_n, where the D_w is the average particle size calculated from the weight-based particle size distribution while D_n is the average particle size calculated from the equivalent number-based particle size distribution). d_{DLS} (nm) is the average size calculated by DLS, and σ_{DLS} is its relative standard deviation. PDI_{DLS} is the dimensionless polydispersity index. D_{TEM} and σ_{TEM} are the diameter and the standard deviation of NPs calculated by TEM. Z-potential (Zpot) and σ_Z are measures of charge between particles and the standard deviation, respectively. pH TA in citrate is the pH of the TA in citrate solution either after thermal preconditioning or at pH 10. pH AgNPs is the pH of AgNP colloidal suspension after synthesis. The size of the AgNPs is compared using the Cohen's d method. In specific, the size and the Z-potential of AgNPs synthesised at [TA] = 0 M is considered a reference to test the effect of TA concentration. The size and the Z-potential at [TA] = 2×10^{-8} M show a big effect size in comparison to the reference (Cohen's d > 0.8)[1].

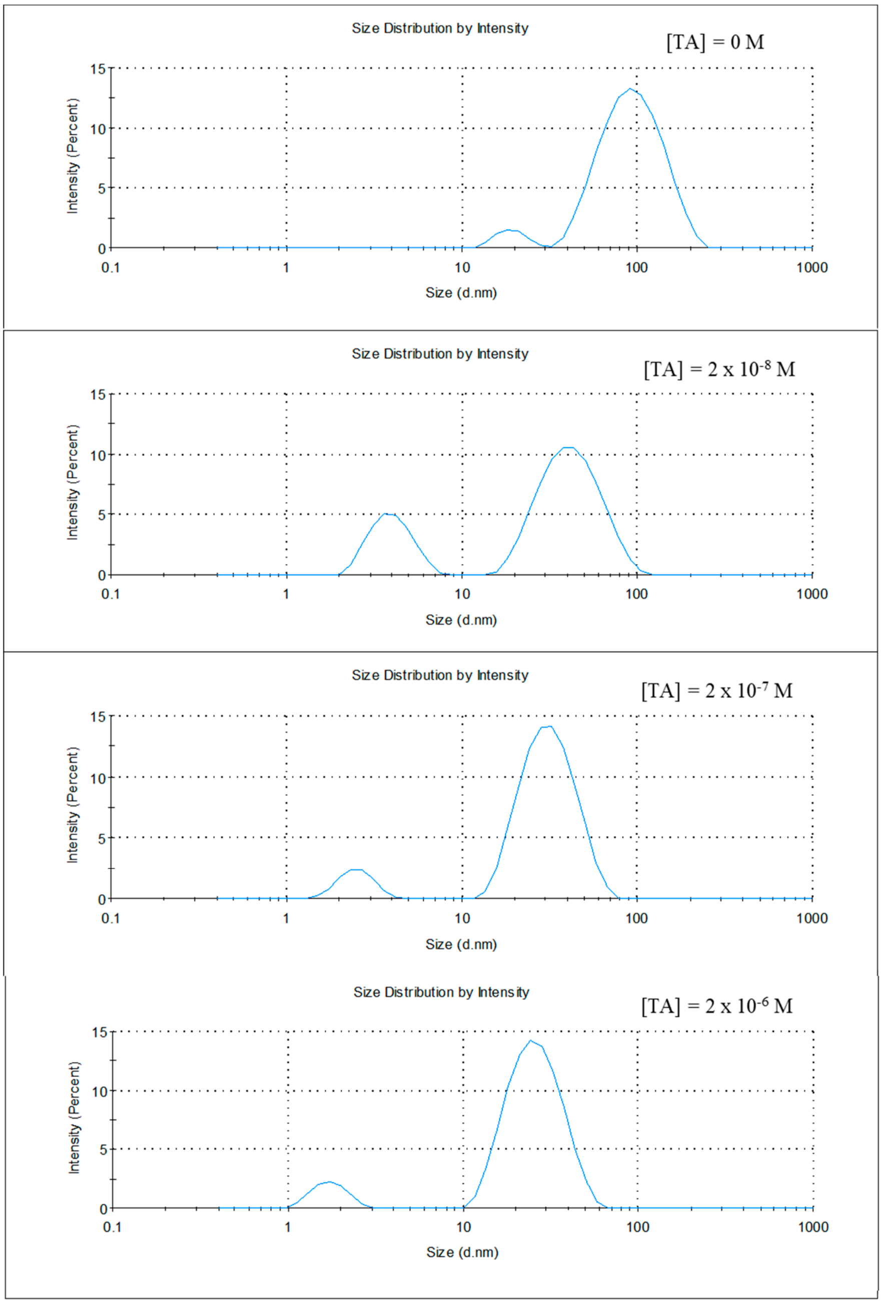

Figure S1. DLS size distribution of AgNPs synthesised by varying the concentration of TA.

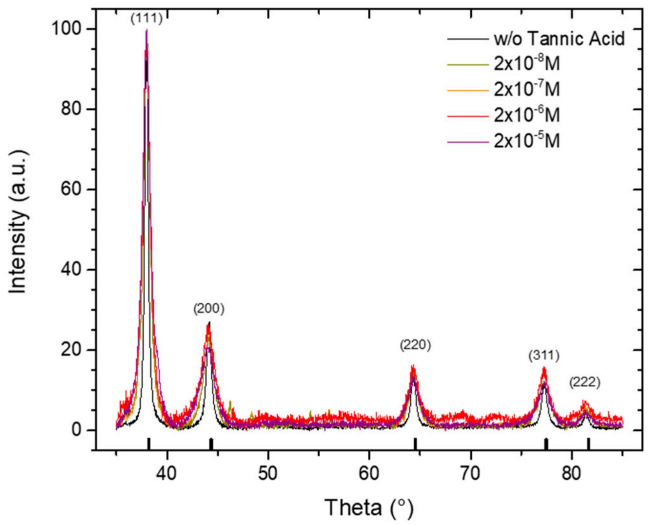

Figure S2. X ray diffractograms of AgNP prepared with different TA concentrations.

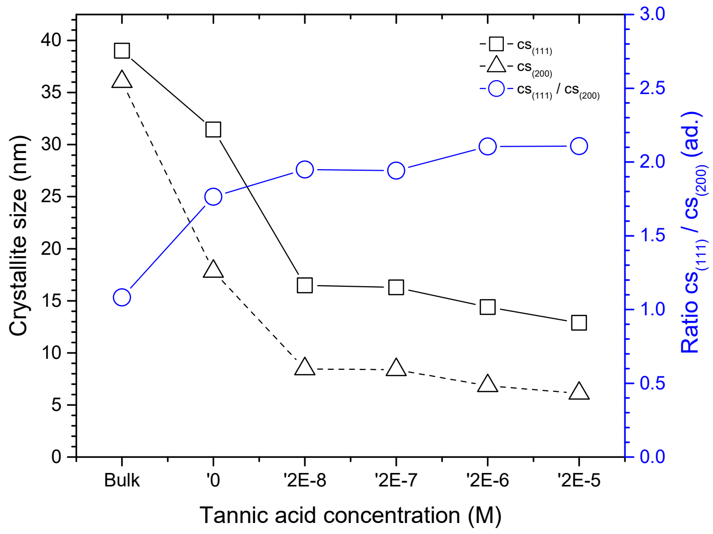

Figure S3. Crystallite size for (111) and (200) facets (left *Y*-axis) as derived from Sherrer's equation and their ratio (right *Y*-axis).

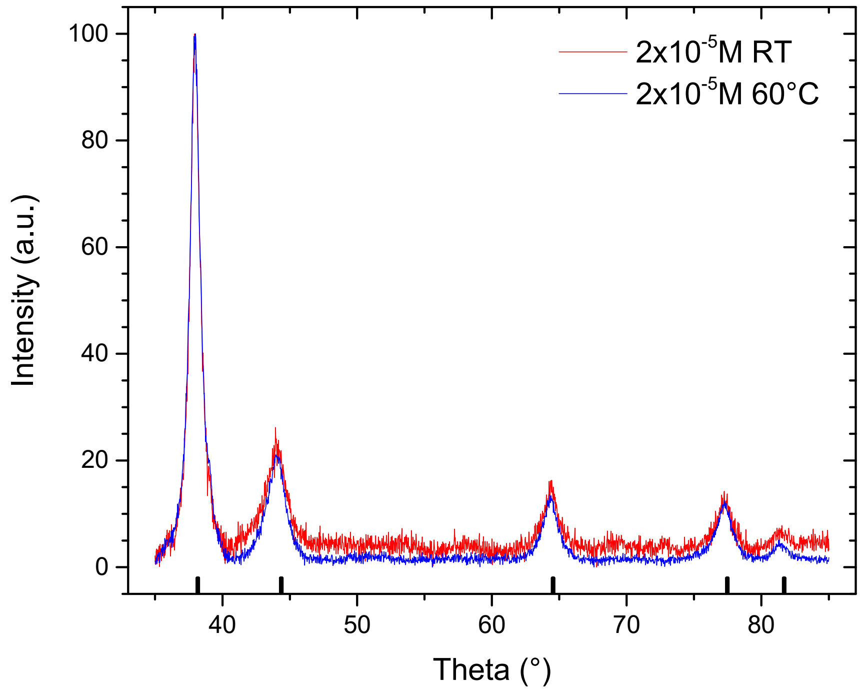

Figure S4. X-ray diffractograms of AgNPs prepared with 2 × 10^{-5} M of TA, which was preconditioned for 45 min.

Table S2. Characterisation of AgNPs synthesised by varying the preconditioning time of TA at 60 °C: the concentration of TA is 2 × 10^{-6} M.

**TA in AgNPs 2 × 10^{-6} M**	**0 min**	**15 min**	**45 min**	**90 min**	**180 min**
$d_{CLS} \pm \sigma_{CLS}$ (nm)	30.0 ± 4.2	25.3 ± 2.8	21.7 ± 2.0	18.3 ± 2.4	18.5 ± 2.4
$DI_{CLS} \pm \sigma_{DI}$	1.17 ± 0.05	1.15 ± 0.02	1.14 ± 0.03	1.21 ± 0.01	1.22 ± 0.03
$d_{DLS} \pm \sigma_{DLS}$ (nm)	14.09 ± 0.07	12.34 ± 0.40	14.00 ± 0.21	18.67 ± 0.10	18.58 ± 0.10
$PDI_{DLS} \pm \sigma_{DLS}$	0.539 ± 0.001	0.525 ± 0.010	0.522 ± 0.008	0.312 ± 0.001	0.329 ± 0.001
$D_{TEM} \pm \sigma_{TEM}$ (nm)	29 ± 9	24 ± 6	19 ± 5	16 ± 5	17 ± 5
Round ± σ_{Round}	0.81± 0.14	0.81 ± 0.14	0.86 ± 0.09	0.88 ± 0.07	0.85 ± 0.09
Z pot ± σ_Z (mV)	−46.7 ± 0.7	−41.7 ± 0.7	−36.8 ± 1.2	−36.1 ± 1.0	−37.1 ± 0.8
pH TA in citrate	7.78	7.74	7.63	7.52	7.54
pH AgNPs	6.38	6.55	6.55	6.53	6.53

d_{CLS} (nm) represents the position of the peak maximum of the AgNP size based on weight distribution, calculated by CLS; σ_{CLS} (nm) is the standard deviation of three independent measurements by CLS calculated at Half Height Width (HHW) of the main peak. DI_{CLS} is the CLS polydispersity index (expressed as the ratio D_w/D_n where the D_w is the average particle size calculated from the weight-based particle size distribution while D_n is the average particle size calculated from the equivalent number-based particle size distribution). d_{DLS} (nm) is the average size calculated by DLS, and σ_{DLS} is its relative standard deviation. PDI_{DLS} is the dimensionless polydispersity index. D_{TEM} and σ_{TEM} are the diameter and the standard deviation of NPs calculated by TEM. Z-potential (Zpot) and σ_Z are measures of charge between particles and the standard deviation, respectively.

Roundness (Round) and σ_{round} are respectively a shape factor calculated by roundness = 4 × area/(π (major axis)^2)), where major axis means the axis of the particle's fitted ellipse and the

standard deviation of roundness. pH TA in citrate is the pH of the TA in citrate solution either after thermal preconditioning or at pH 10. pH AgNPs is the pH of AgNP colloidal suspension after synthesis. pH TA in citrate is the pH of the TA in citrate solution either after thermal preconditioning or at pH 10. pH AgNPs is the pH of AgNP colloidal suspension after synthesis. The size of the AgNPs is compared using the Cohen's d method. In specific, the size and the Z-potential of AgNPs synthesised at [TA] = 2×10^{-6} M without preheating is considered a reference to test the effect of preconditioning time of TA at 60 °C. The size and the Z-potential, after 45 min of preconditioning time of TA at 60 °C, show a big effect size in comparison to the reference (Cohen's d > 0.8).

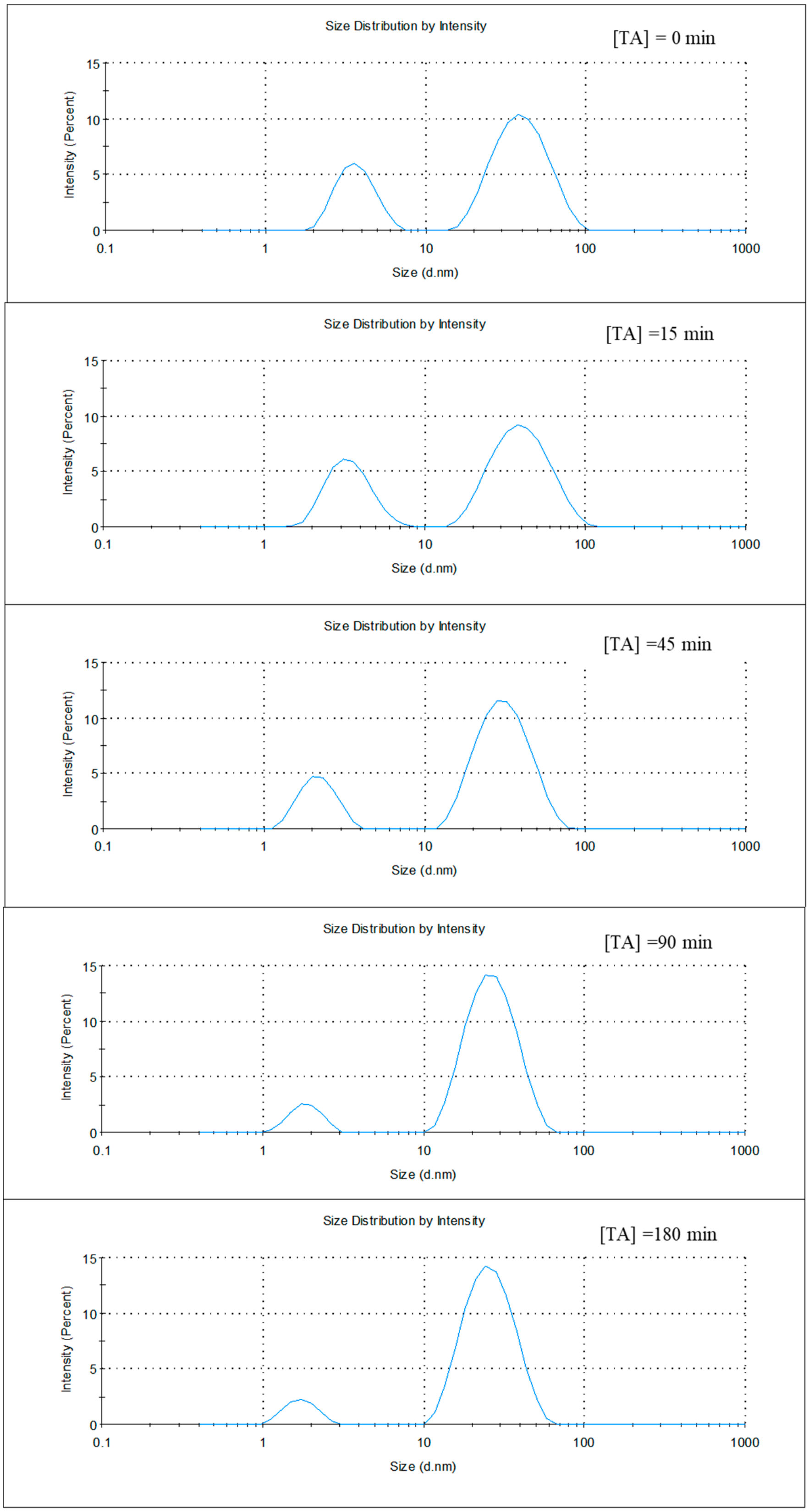

Figure S5. DLS size distribution of AgNPs synthesised by varying the preconditioning time of TA at 60 °C: the concentration of TA is 2×10^{-6} M.

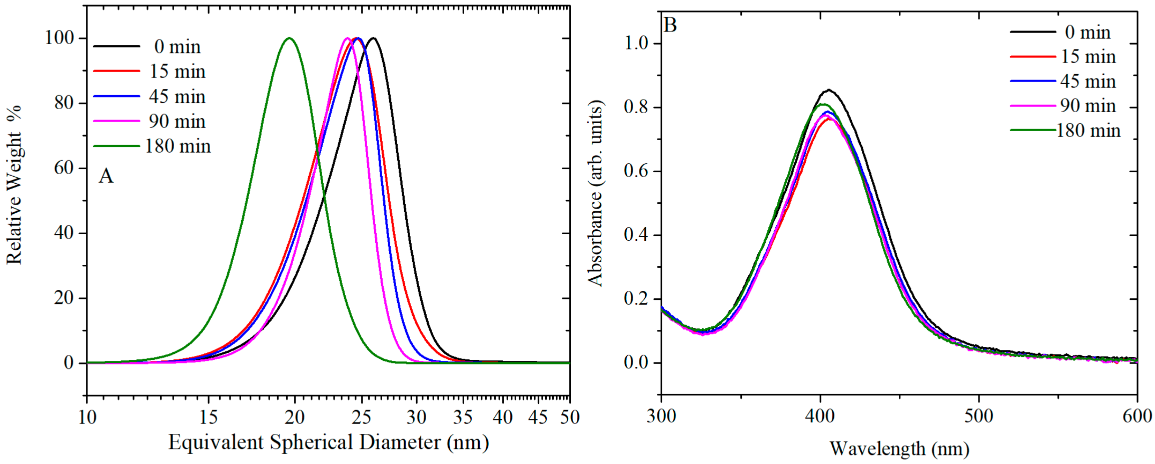

Figure S6. A) CLS size distribution and **B**) UV-vis spectra of AgNPs synthesised with 0.55 mM $AgNO_3$ and 2 × 10^{-5} M of TA concentration by varying the thermal preconditioning time of TA in citrate at 60 °C for 0, 15, 45, 90 and 180 min: CLS and UV-vis spectra show that only at larger preconditioning time of TA (180 min) are AgNPs presented strong size dependence.

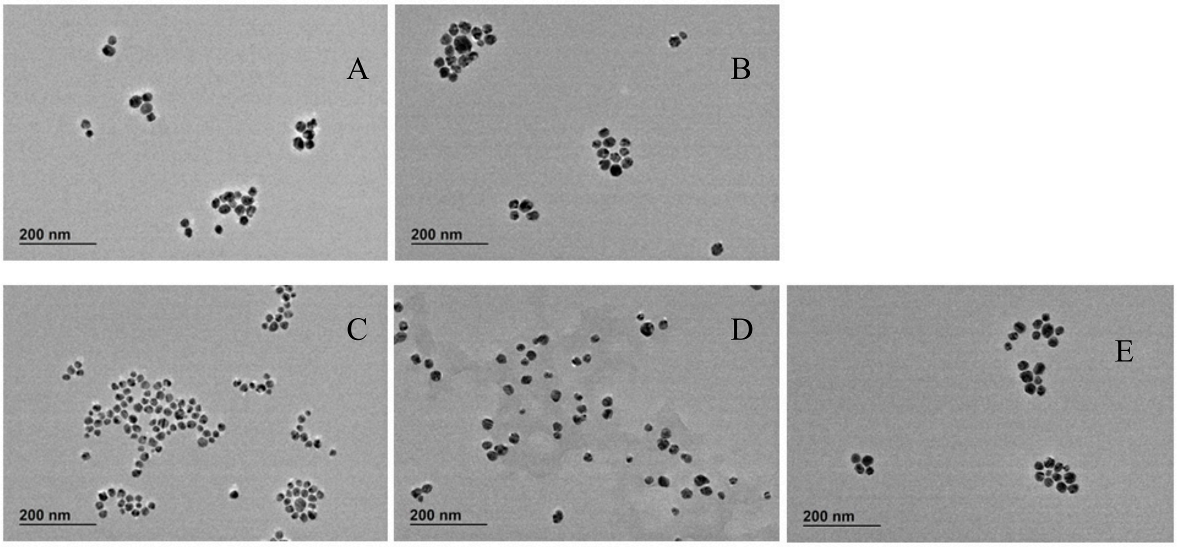

Figure S7. TEM pictures of AgNPs synthesised with 0.55 mM $AgNO_3$ and 2 × 10^{-5} M of TA concentration, varying the thermal preconditioning time of TA in citrate at 60 °C for (**A**) 0 min, (**B**) 15 min, (**C**) 45 min, (**D**) 90 min and (**E**) 180 min.

Table S3. Characterisation of AgNPs synthesised by varying the preconditioning time of TA at 60 °C: the concentration of TA is 2 × 10^{-5} M.

**TA in AgNPs 2 × 10^{-5} M**	**0 min**	**15 min**	**45 min**	**90 min**	**180 min**
$d_{CLS} \pm \sigma_{CLS}$ (nm)	25.3 ± 3.1	23.7 ± 3.1	23.7 ± 2.5	23.0 ± 2.2	18.5 ± 1.9
$DI_{CLS} \pm \sigma_{DI}$	1.11 ± 0.04	1.10 ± 0.02	1.08 ± 0.03	1.06 ± 0.01	1.06 ± 0.01
$d_{DLS} \pm \sigma_{DLS}$ (nm)	25.61 ± 0.08	24.24 ± 0.13	27.71 ± 0.22	28.65 ± 0.34	26.72 ± 0.50
$PDI_{DLS} \pm \sigma_{DLS}$	0.311 ± 0.001	0.308 ± 0.001	0.183 ± 0.001	0.087 ± 0.007	0.05 ± 0.012
$D_{TEM} \pm \sigma_{TEM}$ (nm)	27 ± 5	24 ± 4	24 ± 5	25 ± 5	19 ± 3

Round ± σ_{Round}	0.83 ± 0.08	0.84 ± 0.07	0.83 ± 0.08	0.85 ± 0.07	0.84 ± 0.07
Z pot ± σ_Z (mV)	-47.2 ± 0.8	-47.3 ± 0.8	-47.1 ± 1.19	-44.1 ± 0.8	-43.1 ± 0.9
pH TA in citrate	7.16	7.15	6.94	6.81	6.72
pH AgNPs	7.16	7.15	7.00	6.94	6.72

d_{CLS} (nm) represents the position of the peak maximum of the AgNPs size based on weight distribution, calculated by CLS; σ_{CLS} (nm) is the standard deviation of three independent measurement by CLS calculated at Half Height Width (HHW) of the main peak. DI_{CLS} is the CLS polydispersity index (expressed as the ratio D_w/D_n where the D_w is the average particle size calculated from the weight-based particle size distribution while D_n is the average particle size calculated from the equivalent number-based particle size distribution. d_{DLS} (nm) is the average size calculated by DLS, and σ_{DLS} is its relative standard deviation. PDI_{DLS} is the dimensionless polydispersity index. D_{TEM} and σ_{TEM} are the diameter and the standard deviation of NPs calculated by TEM. Z-potential (Zpot) and σ_Z are measures of charge between particles and the standard deviation, respectively.

Roundness (Round) and σ_{round} are respectively a shape factor calculated by roundness = 4 × area/(π (major axis)^2)), where major axis means the axis of the particle's fitted ellipse and the standard deviation of roundness. pH TA in citrate is the pH of the TA in citrate solution either after thermal preconditioning or at pH 10. pH AgNPs is the pH of AgNP colloidal suspension after synthesis. pH TA in citrate is the pH of the TA in citrate solution either after thermal preconditioning or at pH 10. pH AgNPs is the pH of AgNP colloidal suspension after synthesis. The size of the AgNPs is compared using the Cohen's d method. In specific, the size and the Z-potential of AgNPs synthesised at [TA] = 2 × 10^{-5} M without preheating is considered a reference to test the effect of preconditioning time of TA at 60 °C. The size at any preconditioning time does not shown any statistical difference, whereas the Z-potential, after 45 min of preconditioning time of TA at 60 °C, shows a big effect size in comparison to the reference (Cohen's d > 0.8).

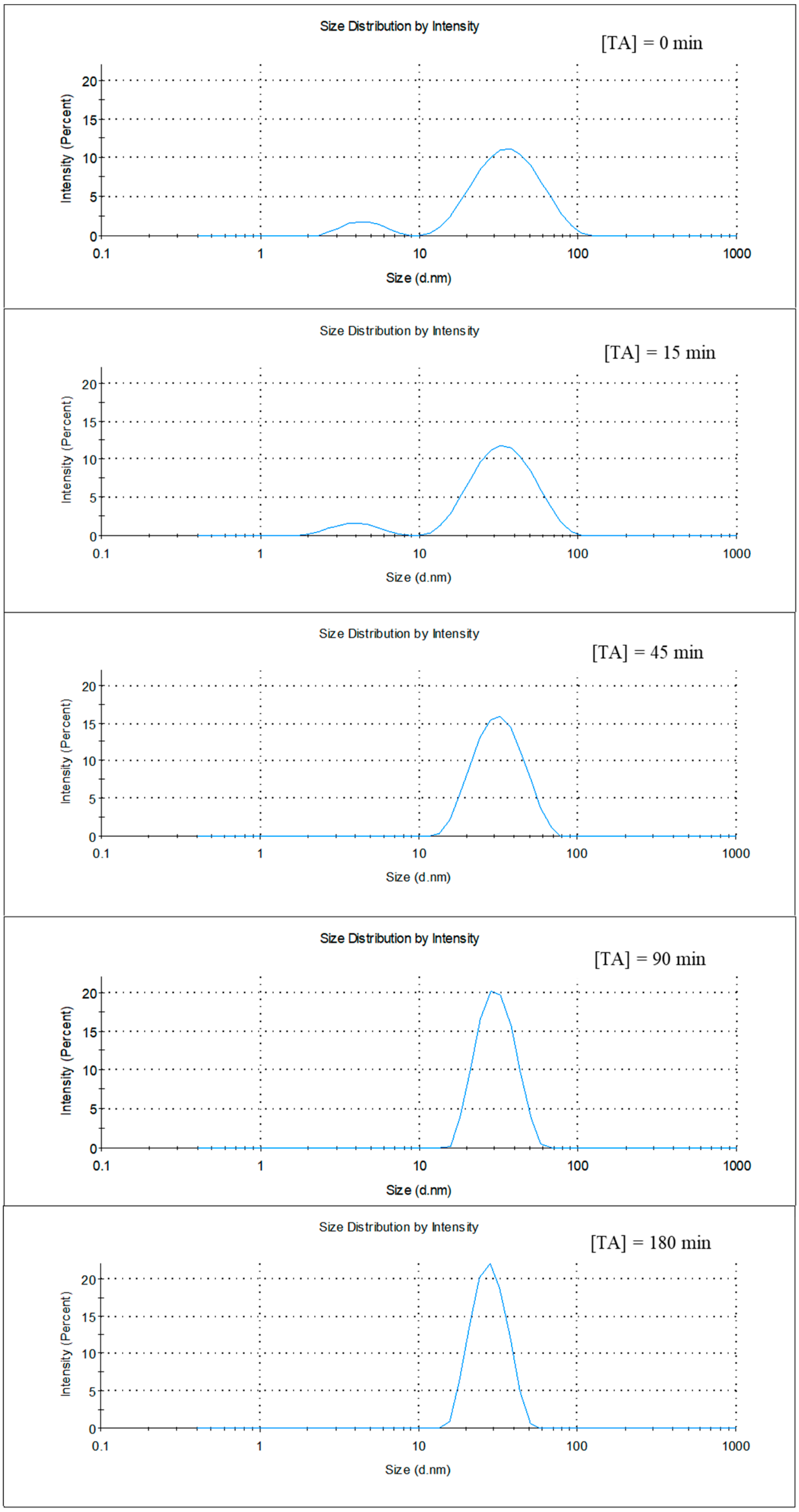

Figure S8. DLS size distribution of AgNPs synthesised by varying the preconditioning time of TA at 60 °C: the concentration of TA is 2×10^{-5} M.

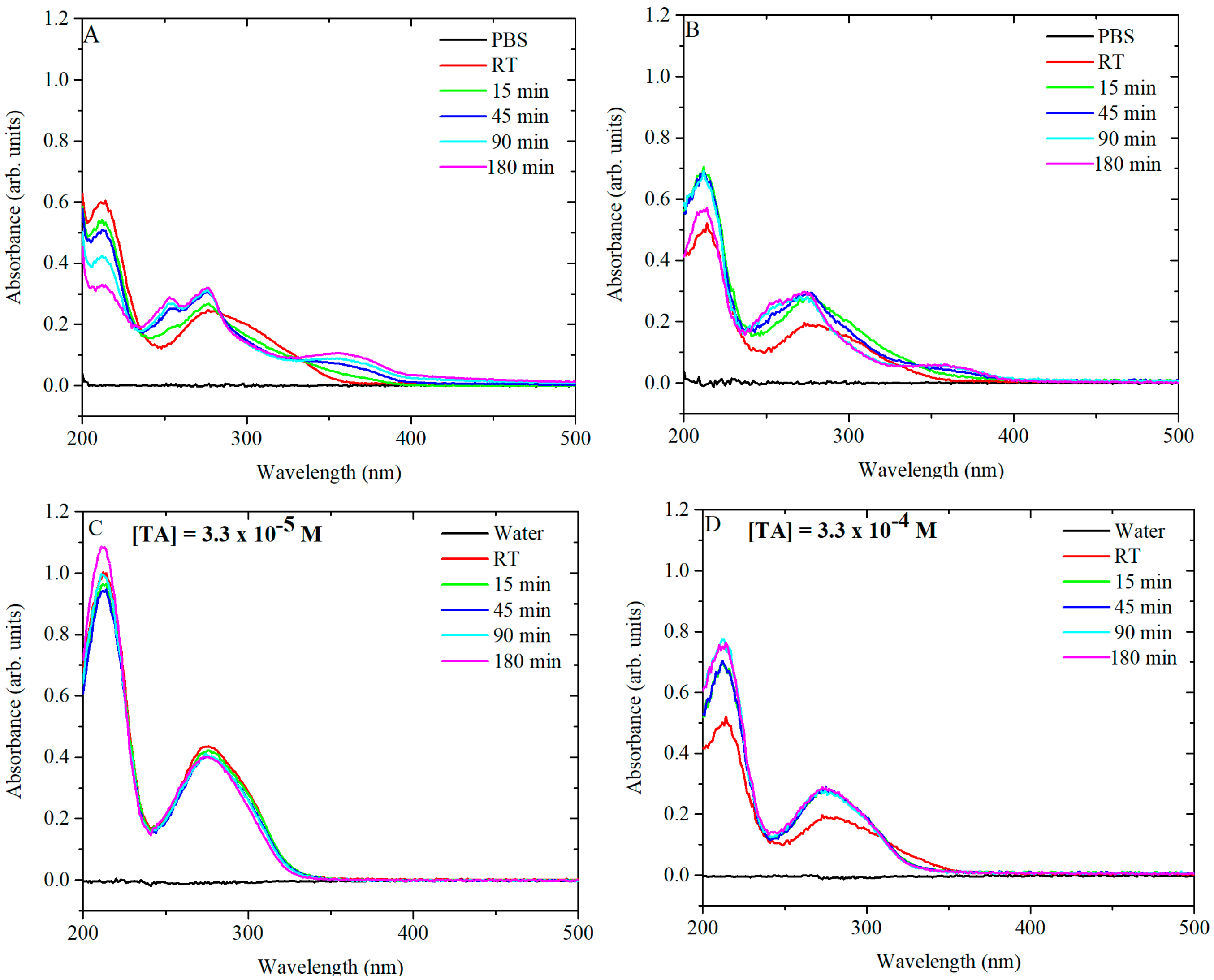

Figure S9. UV-vis spectra of TA solution equilibrated at 60 °C at different time points in phosphate-buffered saline (PBS) and water: the concentration of TA is **A**) 3.3 × 10^{-5} M in PBS, **B**) 3.3 × 10^{-4} M in PBS, **C**) 3.3 × 10^{-5} M in water and **D**) 3.3 × 10^{-4} M in water.

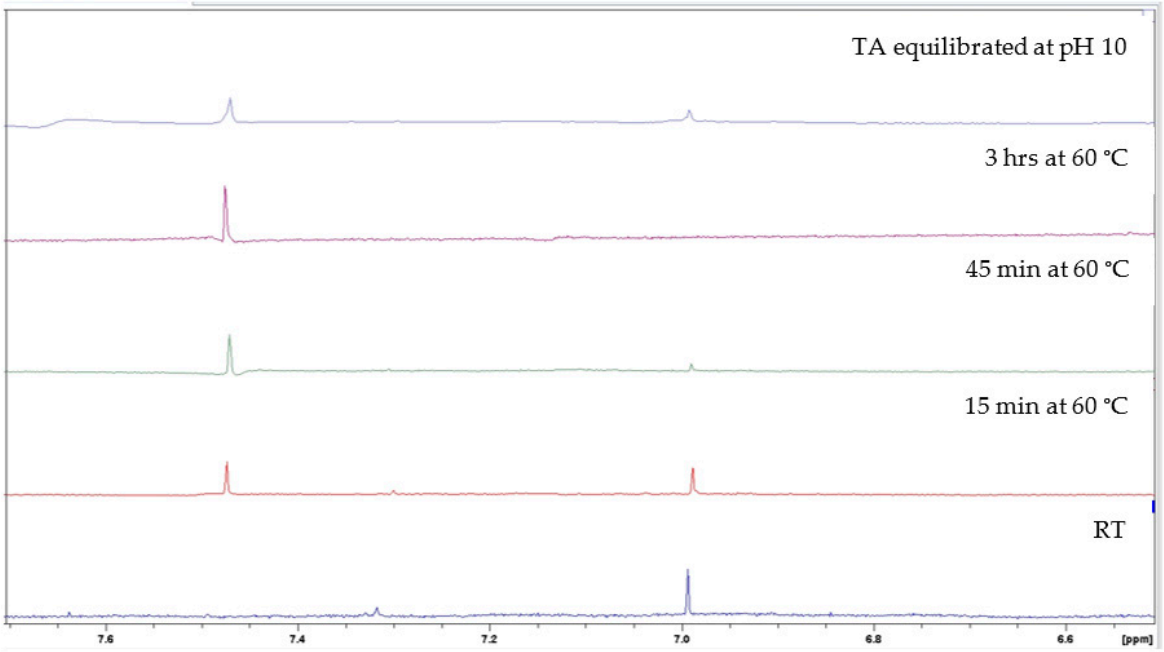

Figure S10. ^{1}H-NMR (zoom of the aromatic part of the spectrum): comparative spectra of TA in PBS by varying the preconditioning time of TA at 60 °C. The concentration of TA is 2×10^{-6} M. Spectra of TA were equilibrated at pH 10.

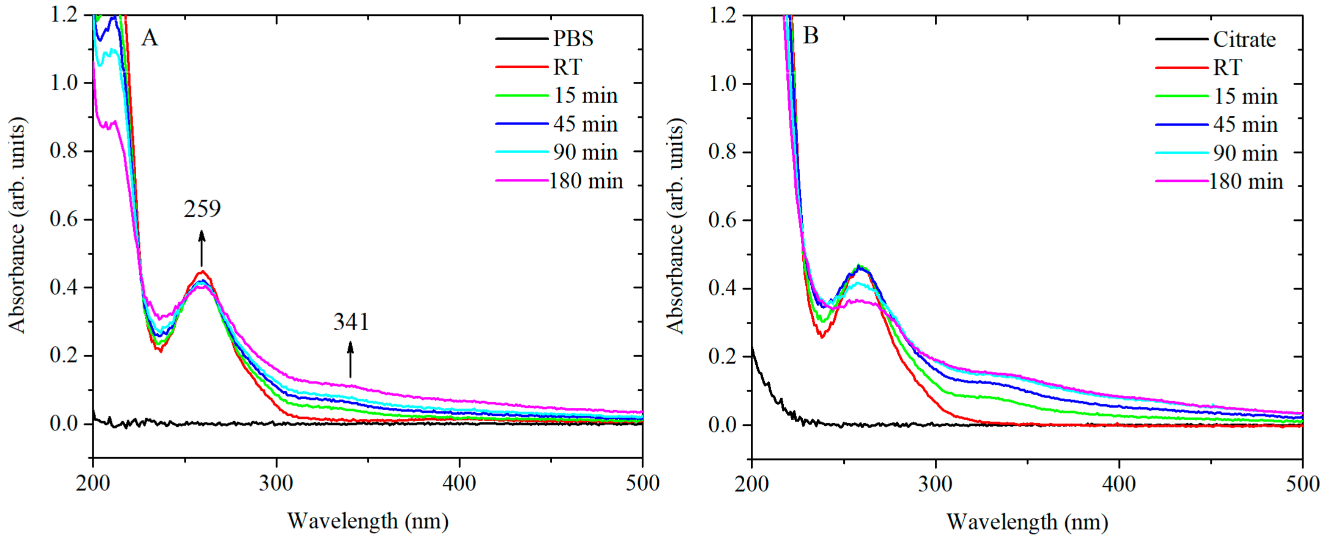

Figure S11. UV-vis spectra of Gallic acid (GA) preconditioned at 60 °C at different time points in **A**) PBS and **B**) citrate.

Table S4. Characterisation of AgNPs synthesised by pH conditioning of TA at pH 10: the concentration of TA is 2×10^{-6} M and 2×10^{-5} M.

[TA] at pH 10	TA 2×10^{-6} M	TA 2×10^{-5} M
$d_{CLS} \pm \sigma_{DLS}$ (nm)	26.2 ± 2.0	18.4 ± 1.6
$DI_{CLS} \pm \sigma_{DI}$	1.12 ± 0.03	1.06 ± 0.03
$d_{DLS} \pm \sigma_{DLS}$ (nm)	11.79 ± 0.07	15.47 ± 0.07
$PDI_{DLS} \pm \sigma_{DLS}$	0.499 ± 0.01	0.459 ± 0.02
$D_{TEM} \pm \sigma_{TEM}$ (nm)	21 ± 6	19 ± 3
Round $\pm \sigma_{Round}$	0.83 ± 0.10	0.86 ± 0.09

Z pot ± σ_Z (mV)	-46.0 ± 0.3	-41.2 ± 0.6
pH TA in citrate	7.79	7.65
pH AgNPs	6.91	6.48

d_{CLS} (nm) represents the position of the peak maximum of the AgNPs size based on weight distribution, calculated by CLS; σ_{CLS} (nm) is the standard deviation of three independent measurements by CLS calculated at Half Height Width (HHW) of the main peak. DI_{CLS} is the CLS polydispersity index (expressed as the ratio D_w/D_n where the D_w is the average particle size calculated from the weight-based particle size distribution while D_n is the average particle size calculated from the equivalent number-based particle size distribution. d_{DLS} (nm) is the average size calculated by DLS, and σ_{DLS} is its relative standard deviation. PDI_{DLS} is the dimensionless polydispersity index. D_{TEM} and σ_{TEM} are the diameter and the standard deviation of NPs calculated by TEM. Z-potential (Zpot) and σ_Z are measures of charge between particles and the standard deviation, respectively.

Roundness (Round) and σ_{round} are respectively a shape factor calculated by roundness = 4 × area/(π (major axis)^2)), where major axis means the axis of the particle's fitted ellipse and the standard deviation of roundness. pH TA in citrate is the pH of the TA in citrate solution either after thermal preconditioning or at pH 10. pH AgNPs is the pH of AgNP colloidal suspension after synthesis. pH TA in citrate is the pH of the TA in citrate solution either after thermal preconditioning or at pH 10.

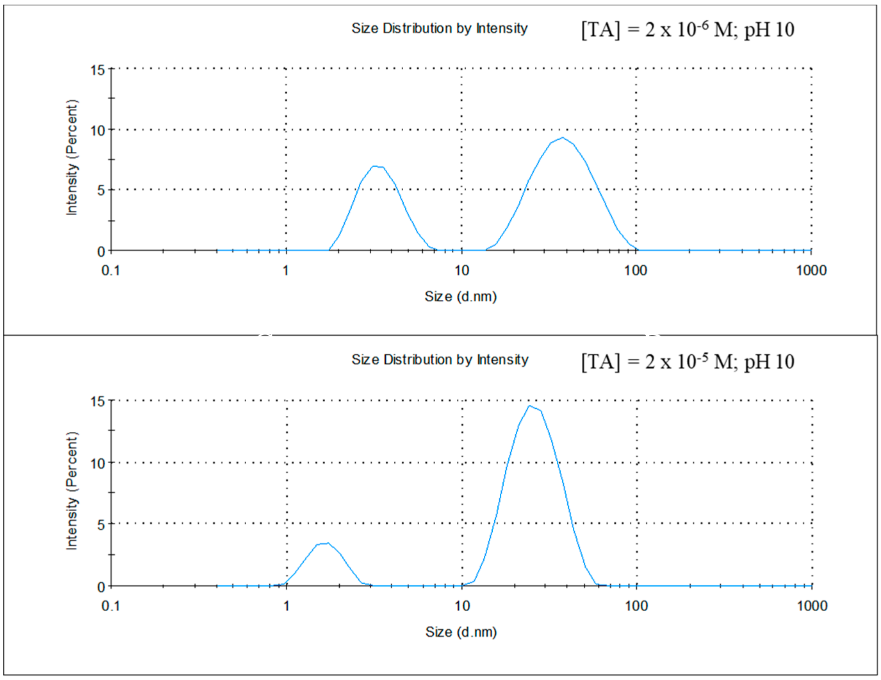

Figure S12. DLS size distribution of AgNPs synthesised by pH conditioning of TA at pH 10: the concentration of TA is 2 × 10^{-6} M and 2 × 10^{-5} M.

References

1. Pellico, J.; Ruiz-Cabello, J.; Saiz-Alía, M.; del Rosario, G.; Caja, S.; Montoya, M.; Fernández de Manuel, L.; Morales, M. P.; Gutiérrez, L.; Galiana, B.; Enríquez, J. A.; Herranz, F. Fast synthesis and bioconjugation of ^{68}Ga core-doped extremely small iron oxide nanoparticles for PET/MR imaging. *Contrast Media Mol. Imaging* **2016**, *11*, 203–210.
